# Meta-analysis of aspirin-guided therapy of colorectal cancer

**DOI:** 10.1007/s00432-022-03942-1

**Published:** 2022-02-16

**Authors:** Johanna C. Mädge, Andreas Stallmach, Lisa Kleebusch, Peter Schlattmann

**Affiliations:** 1 Department of Medical Statistics, Computer Sciences and Data Sciences, Jena University Hospital, 07743 Jena, Germany; 2 Department of Internal Medicine IV, Jena University Hospital, 07743 Jena, Germany; 3 Department of Internal Medicine, Thüringen-Kliniken Pößneck, 07381 Pößneck, Germany

**Keywords:** Aspirin, Colorectal cancer, Survival, Mortality, PIK3CA, PTGS2

## Abstract

**Abstract:**

Purpose colorectal cancer (CRC) is one of the most commonly diagnosed cancers worldwide. Some evidence has shown that aspirin can reduce the morbidity and mortality of CRC. The aim of this meta-analysis was to compare standard care of patients with CRC and standard care with the addition of aspirin in terms of the survival benefit.

**Methods:**

The systematic search was conducted by two independent reviewers in the databases PubMed and Web of Science. Survival data were extracted from studies published before July 2019. We searched for randomised controlled trials, cohort studies and case-control studies.

**Results:**

We included 27 studies in our meta-analysis. There was a sample size of 237,245 patients overall. Aspirin use after diagnosis was associated with an improvement in CRC-specific survival with a hazard ratio (HR) for cancer-related death of 0.74 (95% CI: 0.62–0.89). Our analysis of overall survival data revealed reduced mortality with an HR of 0.82 (95% CI: 0.74–0.90). Patients with the phosphatidylinositol-4, 5-bisphosphate 3-kinase catalytic subunit alpha (PIK3CA) mutation profited from postdiagnosis aspirin use (HR = 0.74, 95% CI: 0.56–0.97). For a high expression of prostaglandin-endoperoxide synthase 2 (PTGS2) = COX-2, we found an HR of 0.65 (95% CI: 0.52–0.82).

**Conclusion:**

Aspirin can improve the outcome of patients with CRC. PIK3CA mutation status and high expression of PTGS2 are associated with longer survival. However, randomised controlled trials are needed to investigate postdiagnosis aspirin use in CRC patients taking into account cancer stage and gene expression.

**Supplementary Information:**

The online version contains supplementary material available at 10.1007/s00432-022-03942-1.

## Introduction

Colorectal cancer (CRC) is one of the most prevalent cancer types around the world. Every year, about 945,000 people are diagnosed—492,000 fatally (Weitz et al. 2005). Due to the high mortality, research into new methods of therapy should be increased. It is still unclear and controversially debated whether nonsteroidal anti-inflammatory drugs (NSAIDS)—acetylsalicylic acid (aspirin) in particular—have an influence on the development of CRC and whether they could be used for primary prevention of CRC (Bosetti et al. 2012). A recent meta-analysis (Haykal et al. 2019) did not find a reduction of cancer-related mortality and no reduced incidence of CRC. In recent years, randomised controlled trials have been used to investigate whether aspirin can help as an additional therapy after a diagnosis of CRC and to show whether patients have better outcomes than those receiving standard therapy (Michel et al. 2018). It is unclear whether the outcome depends on the time of starting aspirin, i.e., comparing patients who were taking aspirin before their diagnosis for other reasons (e.g., cardiovascular risk factors) and continued afterwards (primary prevention of CRC combined with tertiary prevention) with those who only began taking aspirin after their diagnosis of CRC (tertiary prevention). Furthermore, it is not clear whether certain gene expression types have an influence on the outcome of CRC. It was recently shown that the gene phosphatidylinositol-4, 5-bisphosphate 3-kinase catalytic subunit alpha (PIK3CA) and its mutation could be associated with patient survival (Domingo et al. 2013). Wu et al. (Wu et al. 2013) conducted a meta-analysis and demonstrated that PIK3CA mutation is associated with poor survival for patients with metastatic CRC. If there is an association between gene expression and outcome, further research could help to find new therapies for CRC.

Aspirin’s mechanism of action is based on the irreversible inactivation of cyclooxygenase, a prostaglandin oxidase reductase, with two isoenzymes: COX-1 and COX-2. Due to the suppression of prostaglandin and thromboxane, it has effects on platelet aggregation and inflammation. Reducing the prostaglandin E2 (PGE2) production of COX-2 can decrease tumour cell proliferation by different pathways. COX-2 is therefore also known as prostaglandin-endoperoxide synthase 2 or PTGS2. The inhibition of COX-1 in platelets results in a lower production of thromboxane A2 (TXA2) and vascular endothelial growth factor (VEGF), which reduces angiogenesis and metastasis of the tumour. Additionally, aspirin inhibits the nuclear translocation of nuclear factor kappa-light-chain-enhancer of activated *B* cells (NF-κB) and supports apoptosis of tumour cells by that pathway. It also induces apoptosis by affecting the ratio of B-cell lymphoma protein 2-associated X (Bax) to B-cell lymphoma protein 2 (Bcl-2). Moreover, it was found that aspirin enhances the expression of death receptor 5 (DR5), which yields another way to increase apoptosis. By inhibiting the mammalian target of rapamycin (mTOR) and activating AMP-activated protein kinase (AMPK), aspirin has a positive effect on autophagy in CRC cell models. In addition, aspirin stimulates the DNA mismatch repair (MMR) system and has been reported to suppress oxidative stress (Ma et al. 2017). Overexpression of COX-2 has been found in CRC cells, which might be an important point of action (Di Francesco et al. 2015). It is still a subject of debate whether or not aspirin has the potential to reduce the incidence of CRC (Bosetti et al. 2012). Due to negative side effects such as gastrointestinal bleeding, it is not recommended as primary prevention. According to many clinical studies published in the last few years, aspirin is becoming more important as a new therapy of CRC. Today, the standard care in CRC consists of surgery, radiotherapy and chemoradiotherapy. Furthermore, fluorouracil-based adjuvant chemotherapy is recommended for patients with stage III colon cancer which has been completely resected or high-risk stage II colon cancer (Weitz et al. 2005).

One side effect of aspirin therapy is gastrointestinal bleeding (Haykal et al. 2019). We evaluated relevant bleeding events described in the studies in this review, and we highlighted the advantages and disadvantages of aspirin. Against this background, we conducted a meta-analysis to present the current state of aspirin-guided CRC therapy.

## Materials and methods

### Literature search and study selection

A protocol for the systematic search strategy was prepared in advance by J.C.M. and P.S. This included explanations of the study synopsis, the medical problem, design aspects, statistical analysis and information synthesis. The Preferred Reporting Items for Systematic Reviews and Meta-Analyses (PRISMA) statement was used to report the results (Moher et al. 2009). The systematic search was carried out by two independent reviewers in the databases PubMed and Web of Science. The former was conducted by J.C.M. and L.K. In case of any disagreement, the matter was discussed with P.S., and then, a consensus was found. The literature sources were managed with Endnote. We included relevant studies published before July 2019. Titles and abstracts were scanned, and where appropriate, the full papers were read and the inclusion criteria evaluated. For our analysis, we searched for randomised controlled trials, because they are the preferred design for studying effects and they are more likely to provide unbiased information than observational study designs (Reeves et al. 2020). Additionally, cohort studies and population-based case-control studies were accepted.

The following keywords were used: “Aspirin” AND “Colon cancer”; “Aspirin” AND “Colon cancer” AND “Prognosis”; “Aspirin” AND “Colon cancer” AND “Outcome”; “Aspirin” AND “Colon cancer” AND “postoperative”. Those keywords were selected, because patients with CRC and aspirin therapy were prerequisites for inclusion and because we wanted to clarify the results with additional criteria that would yield outcome data regarding the prognoses of patients who mostly underwent surgery. The search strategy addressed the influence of different genes on patient survival including PIK3CA. The investigation of other genes, such as PTGS2 (COX-2), depended on the availability of data in the respective studies. Uncontrolled studies and review articles were excluded from the results. Only studies with new primary data were included. Furthermore, some results of the literature search were excluded after the full texts were read in case where no patient survival data were available. As an expert in gastroenterology, A.S. was involved in the process of finding unpublished literature. After having finished the search, we also scanned interesting reviews and studies for missed matching studies.

### Data extraction

Based on a structured data extraction sheet, data were extracted and subsequently compared, and disagreements were resolved. The following items were deemed to be relevant: study ID, citation, design, duration, blinding, number and characteristics of participants, interventions, outcomes/results, adverse outcomes, instruments/scales applied, relative risk, and odds ratios with the corresponding error or 95% confidence intervals. The Cochrane Collaboration’s tool for assessing risk of bias was used. The Newcastle Ottawa Scale was used to evaluate study quality (Stang 2010).

### Statistical analysis

Published hazard ratios (HR) and odds ratios from case-control studies were combined using the inverse variance method. Heterogeneity was measured using Cochrane’s *Q* test, the *I*^2^ measure, and the heterogeneity variance based on the random effects model according to DerSimonian and Laird (DerSimonian and Laird 1986). Hazard ratios were extracted with their corresponding 95% confidence intervals. Since a meta-analysis is an observational study, the statistical analysis covered the investigation of bias, chance, and confounding. We classified each study for its level of evidence.

The results were presented graphically in a forest plot. Forest plots were presented by intervention. Other descriptive measures include confounder variables, means of statistical analysis, study design and publication year or performing year.

At the first stage, the data were visualised with a funnel plot which is a scatter plot of sample size and effect sizes. A more formal analysis of publication bias (i.e., file-drawer bias which refers to the possibility that only positive studies are published especially in the case of small studies) was based on Egger’s test or other appropriate methods (Deeks et al. 2005). A summary HR was determined together with a 95% confidence interval. The cut-off for statistical significance was set at *p* < 0.05. Additionally, all estimates were separated by intervention. In this meta-analysis we used the random effects model to calculate the global result of the effect measures of each study (Schwarzer 2015). If no heterogeneity was present, a fixed-effects model was applied. Subgroup analyses were added for different gene expressions in patients. Cochrane’s *Q* test was applied as an initial test for heterogeneity. Based on this test, the percentages of total variation across studies which are due to heterogeneity rather than chance (I^2^) were estimated and presented. Calculations were performed with R 3.6.3 (R Core Team 2019) and the package meta (Balduzzi et al. 2019). For all studies which are not randomised controlled trials (RCTs), we investigated age, sex, stage, aspirin dosage, and different gene expressions of the given population. The analysis was redone by leaving out one study for sensitivity analysis.

## Results

### Studies selected for the review

In total, we selected 27 studies in our meta-analysis which fulfilled the inclusion criteria. Two studies were case-control studies and the remaining 25 were cohort studies. Due to the low number of case-control studies, we evaluated them together with the cohort studies as a single group. No completed randomised controlled study was found. For a few studies, it was difficult to obtain the full texts. The local university library was consulted for help. For some publications, especially very old ones, the full texts were located in this way. The remaining results without a successful full-text search were excluded. During the literature search, which included randomised controlled studies, we found several study protocols of ongoing studies.

The total sample size was 237,245 patients. Of the selected studies, 19 were used for the analysis of postdiagnosis aspirin use and 12 for the analysis of aspirin use before CRC diagnosis. The included studies were published between 2009 and 2017. Further information, such as study design, country, aspirin dosage, sample size and adjustments, and those regarding age, sex, cancer type, and stage can be found in Table 1 ‘Study characteristics’ and its complete version (Supplementary, Table 2). We extracted data for overall-, recurrence-free- and CRC-specific survival of patients with CRC. Furthermore, we categorised the data into two groups: the patients with CRC who were taking aspirin before their diagnosis (e.g., for cardiovascular prevention), hereafter called ‘prediagnosis Aspirin use’, and the patients who used aspirin after their diagnosis of CRC (‘postdiagnosis Aspirin use’). Additionally, in the postdiagnosis aspirin use group, we made a subgroup analysis of certain gene expressions. For that, we had a look at PIK3CA and PTGS2. We did not find a publication bias in our study with the use of the command ‘metabias’ in R. Furthermore, all subgroup analyses were repeated by leaving out one study. For that, we used the function ‘metainf’ in R. We did not find any study which stood out. Study quality was assessed using the Newcastle Ottawa Scale (Stang 2010). This scale tries to address the quality of studies which are not randomised and controlled by the use of three categories. These are ‘selection’, ‘comparability’ and ‘outcome’ for cohort studies or ‘selection’, ‘comparability’ and ‘exposure’ for case-control studies. The maximum number of points on the scale is 9. The results are presented in Table 1. In conclusion, most of the studies were graded as high quality and the scores ranged between 6 and 9. They did not have relevant shortcomings. Both of the case-control studies (Cardwell et al. 2014; Din et al. 2010) had lower scores of 4 and 5 points.

**Table 1:** Study characteristics (for full data sheet, see Supplementary Table 2)

Study	Year	Sample size	Gene analysis	Dose (mg)	pre-/postdiagnosis	Cancer type	Stage	Outcome	Study quality
Bains et al. (2016)	2016	23,162	None	75/160	Post	CRC	I–IV	All-cause deaths: 9289, CRC-specific deaths: 6533	9
Bastiaannet et al. (2012)	2012	4481	None	80/30	Post	CRC	I–IV	n.a.	8
Cardwell et al. (2014)	2014	12,868	None	25 (0.3%)/75 (98.5%)/ > 300 (1.2%)	Pre + post	CRC	I–IV	All-cause deaths: 2214, CRC-specific deaths: 1559	4
Chan et al. (2009)	2009	1279	None	325	Pre + post	CRC	I–III	All-cause deaths: 480, CRC-specific deaths: 222	7
Domingo et al. (2013)	2013	896	PIK3CA	< 100	Post	CRC	II–III	All-cause deaths: 395	8
Frouws et al. (2017)	2017	599	BRAF, KRAS	80–100	Post	CC	I–IV	All-cause deaths: 267	8
Goh et al. (2014)	2014	726	None	100	Pre + post	CRC	I–III	CRC-specific deaths: 181	8
Gray et al. (2017)	2017	680	PTGS2, PIK3CA	75	Post	CRC	II–III	All-cause deaths: 299, CRC-specific deaths: 212	8
Hamada et al. (2017)	2017	617	CD274	81/325	Post	CRC	I–IV	All-cause deaths: 325, CRC-specific deaths: 118	6
Hua et al. (2017)	2017	2419	KRAS, BRAF	n.a.	Pre + post	CRC	I–IV	All-cause deaths: 381, CRC-specific deaths: 100	8
Liao et al. (2012)	2012	964	PIK3CA, KRAS, BRAF, PTGS2, CIMP, LINE-1, phosphorylated AKT	325	Post	CRC	I–IV	All-cause deaths: 395, CRC-specific deaths: 190	6
McCowan et al. (2013)	2013	2990	None	75/300	Pre + post	CRC	I–IV	All-cause deaths: 1998, CRC-specific deaths: 1021	9
Ng et al. (2015)	2015	799	None	n.a.	Post	CC	III	All-cause deaths: 156	6
Reimers et al. (2014)	2014	999	PTGS2, PIK3CA, HLA class I	75–325	Post	CC	I–IV	All-cause deaths: 465	8
Walker et al. (2012)	2012	13,944	None	75/ > 75	Pre + post	CRC	I–IV	All-cause deaths: 5358	8
Coghill, et al. (2011a, b)	2011	1737	None	n.a.	Pre	CRC	I–IV	All-cause deaths: 707, CRC-specific deaths: 262	8
Coghill, et al. (2011a, b)	2011	1051	None	n.a.	Pre	CRC	I–IV	All-cause deaths: 371, CRC-specific deaths: 274	9
Coghill et al. (2012)	2012	160,143	None	< 200- > 325 mg	Pre	CRC	I–IV	All-cause deaths: 15,608, CRC-specific deaths: 492	7
Din et al. (2010)	2010	2259	None	75	Pre	CRC	I–IV	All-cause deaths: 670, CRC-specific deaths: 561	5
Giampieri et al. (2017)	2017	66	KRAS, BRAF	100	Pre	CRC	I–III	All-cause deaths: 66	8
Hippisley-Cox and Coupland (2017)	2017	44,145	None	n.a.	Pre	CRC	I–IV	All-cause deaths: 26,887, CRC-specific deaths: 13,588 (derivation cohort)	8
Kim et al. (2015)	2015	686	None	n.a.	Pre	CRC	III	n.a.	8
Kothari et al. (2015)	2015	1487	PIK3CA	81–325	Pre	CRC	I–IV	n.a.	8
Murphy et al. (2017)	2017	488	PIK3CA	> 75	Post	CC	II	All-cause deaths (PIK3CA-Mutation): 17; all-cause deaths (PIK3CA-Wildtype.): 80	8
Zell et al. (2009)	2009	621	None	n.a.	Pre	CRC	I–IV	all-cause deaths: 222, CRC-specific deaths: 145	7
Zanders et al. (2015)	2015	1043	None	< 100	Post	CRC	I–IV	All-cause deaths: 494	9
Restivo et al. (2015)	2015	241	None	100	Post	RC	II–III	n.a.	8

### Postdiagnosis aspirin use

**Fig. 1:**
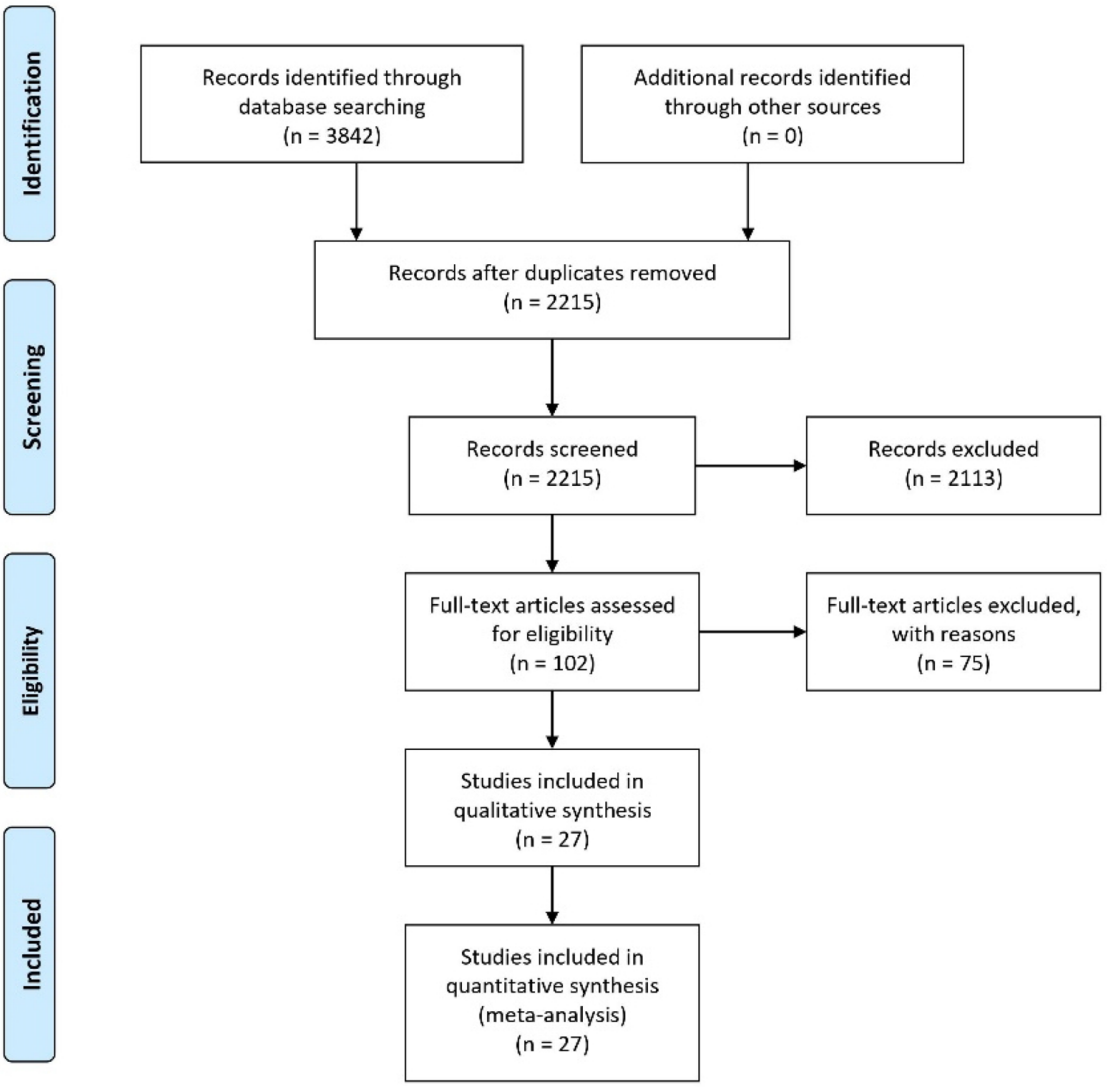
PRISMA flow diagram (Moher et al. 2009)

**Fig. 2:**
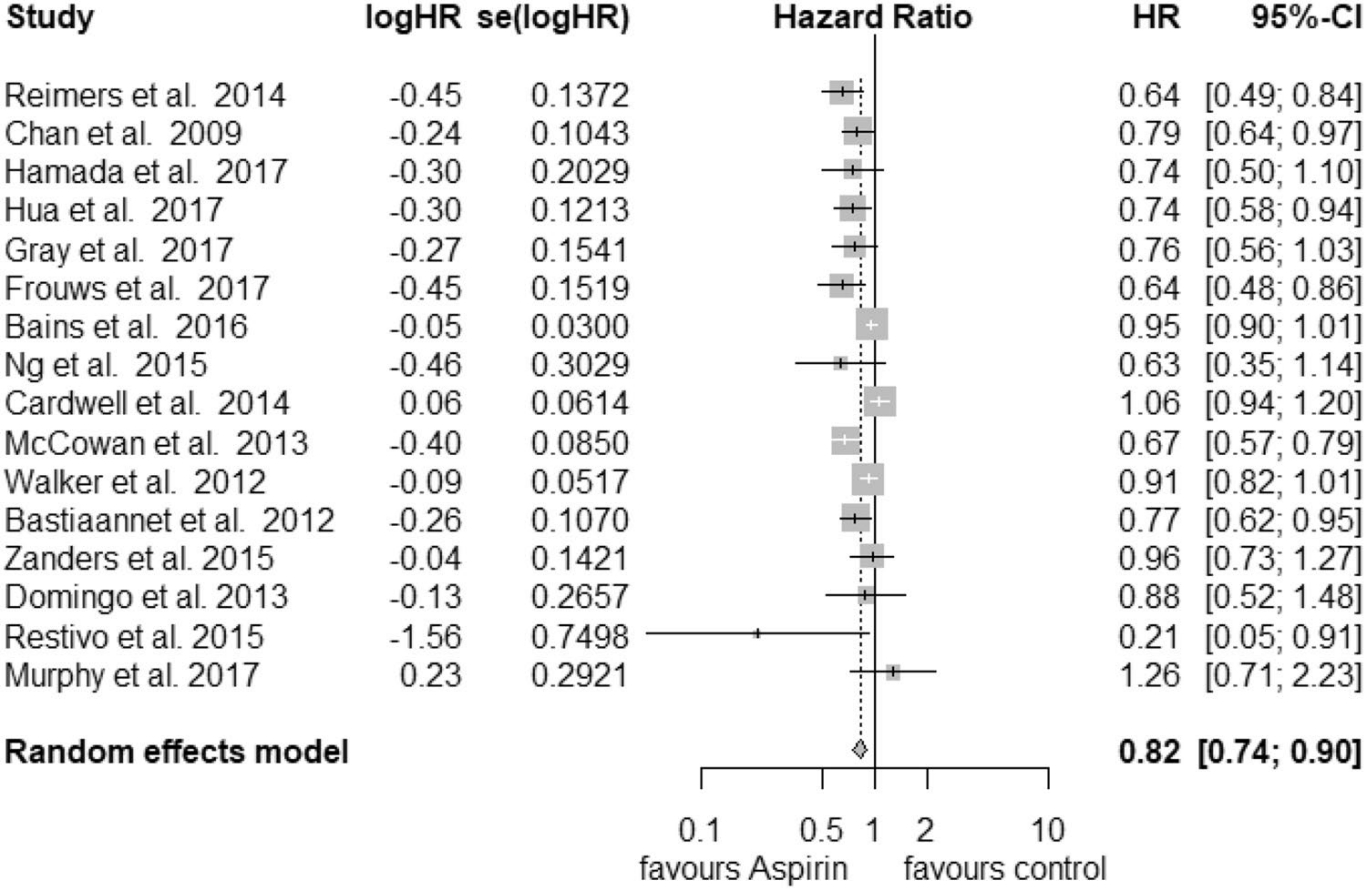
Postdiagnosis aspirin use, overall survival

**Fig. 3:**
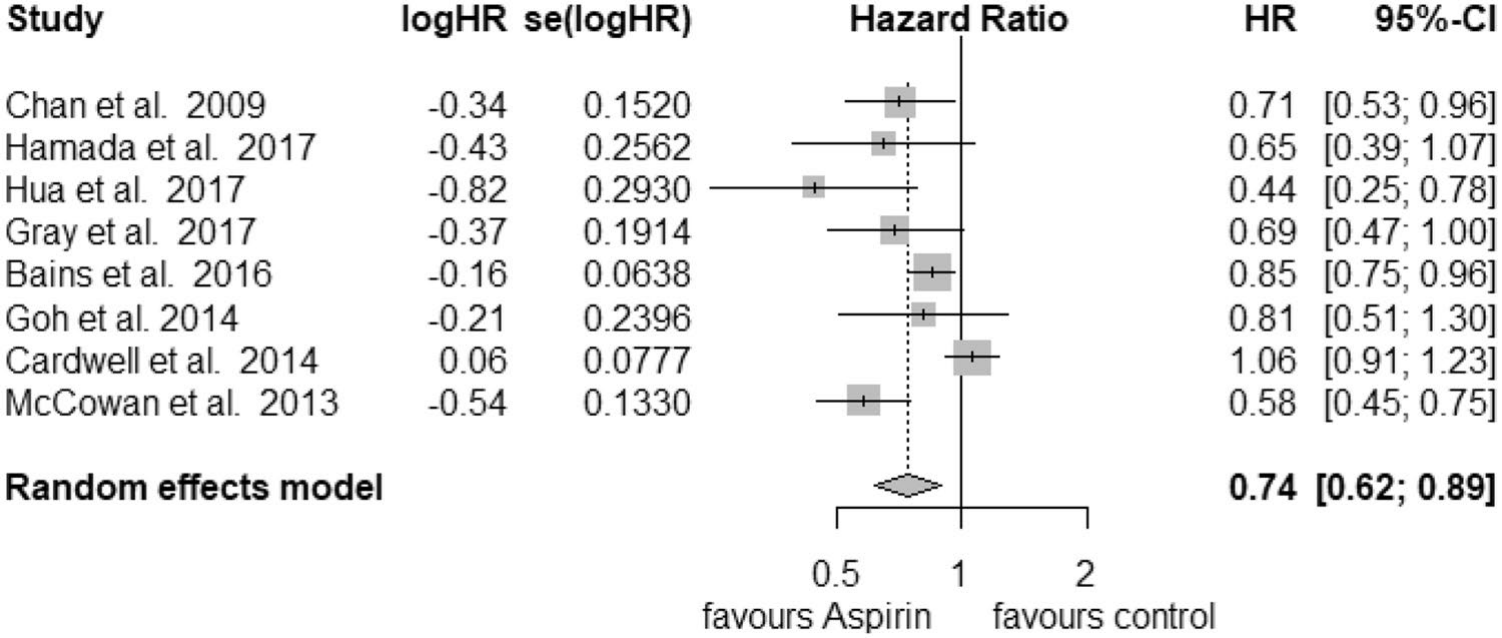
Postdiagnosis aspirin use, CRC-specific survival

In total, 18 studies were used in the analysis of postdiagnosis aspirin use (including PIK3CA and PTGS2 analysis) (Fig. 1). Of those, 8 were included for CRC-specific survival, 3 for recurrence-free survival and 16 for overall survival calculations. We found an improvement of CRC-specific survival (Fig. 2) with a hazard ratio (HR) for cancer-related death of 0.74 (95% CI: 0.62–0.89) and a substantial heterogeneity (*I*^2^ = 72%, tau^2^ = 0.0425, *Q* = 24.89, *p* < 0.01). We did not find publication bias (*t* = − 2.21, *p* = 0.07). Our analysis of overall survival (Fig. 3) in the postdiagnosis aspirin use group revealed a lower mortality rate, based on an HR of 0.82 (95% CI: 0.74–0.90). The heterogeneity amounted to *I*^2^ = 69% (tau^2^ = 0.0191, *Q* = 48.66, *p* < 0.01). However, publication bias cannot be ruled out (*t* = − 2.78, *p* = 0.015). We then analysed recurrence-free survival data (see Supplementary Fig. 8). Here, we found reduced mortality with an HR of 0.50 (95% CI: 0.33–0.76) with a heterogeneity of *I*^2^ = 0% (tau^2^ = 0, *Q* = 0.8, *p* = 0.67). Therefore, we used the fixed-effects model. No publication bias was found (*t* = − 0.34, *p* = 0.79).

### Prediagnosis aspirin use

The analysis of prediagnosis aspirin use was based on 14 studies, 12 of which were used for CRC-specific survival and nine of which for overall survival. We should print out that for both subgroups, Hippisley-Cox et al. (Hippisley-Cox and Coupland 2017) distinguished between men and women. Those were assessed as two separate studies. An HR of 0.91 (95% CI: 0.82–1.01) showed an improved overall survival and CRC-specific survival (see Supplementary Figs. 9 and 10). Heterogeneity for CRC-specific survival was calculated as *I*^2^ = 71% (tau^2^ = 0.0203, *Q* = 40.99) with a statistical significance of *p* < 0.01. There was no evidence of publication bias (*t *= 1.17, *p* = 0.27). For overall survival, we found a considerable heterogeneity of *I*^2^ = 77% (tau^2^ = 0.0147, *Q* = 39.51, p < 0.01); no publication bias was detected (*t *= − 0.31, *p* = 0.77).

### PIK3CA

**Fig. 4:**
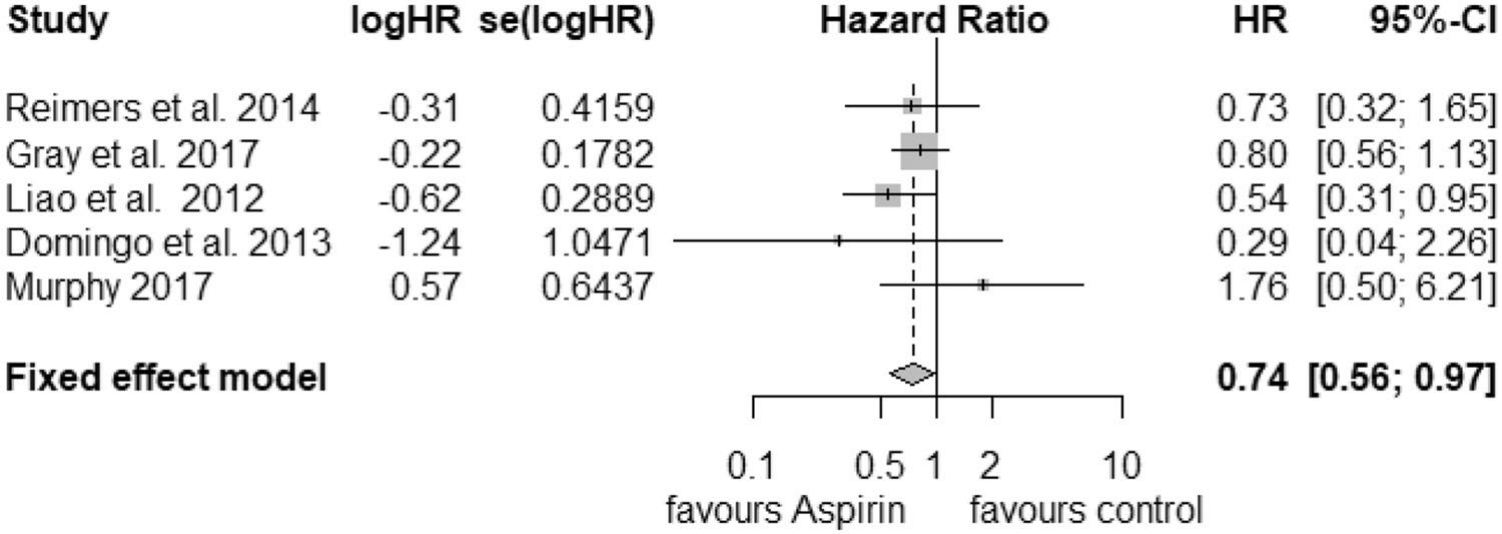
Postdiagnosis aspirin use, PIK3CA mutation

**Fig. 5:**
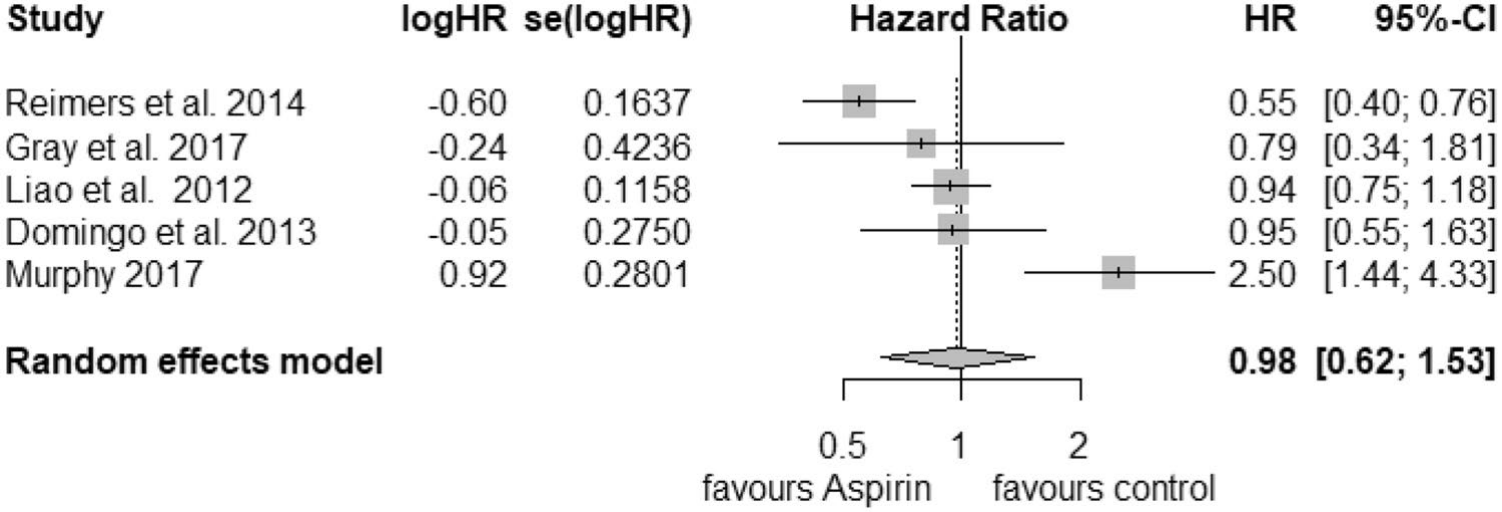
Postdiagnosis aspirin use, PIK3CA wild-type

For the analysis of postdiagnosis aspirin use regarding the PIK3CA mutation status, we found 5 corresponding studies. The pooled HR for cancer-related death considering PIK3CA-mutated tumours (Fig. 4) was calculated as 0.74 (95% CI: 0.56–0.97) with a heterogeneity of *I*^2^ = 0% (tau^2^ = 0, *Q* = 3.99, *p* = 0.41). For that reason, we selected the fixed-effects model. Publication bias was not found (*t* = − 0.15, *p *= 0.89). PIK3CA wild-type status (Fig. 5) was associated with an HR of 0.98 (95% CI: 0.62–1.53) and a considerable heterogeneity of *I*^2^ = 82% (tau^2^ = 0.2013, *Q* = 22.6) and statistical significance of *p* < 0.01. We did not find publication bias (*t *= 0.51, *p* = 0.64).

### PTGS2

**Fig. 6:**
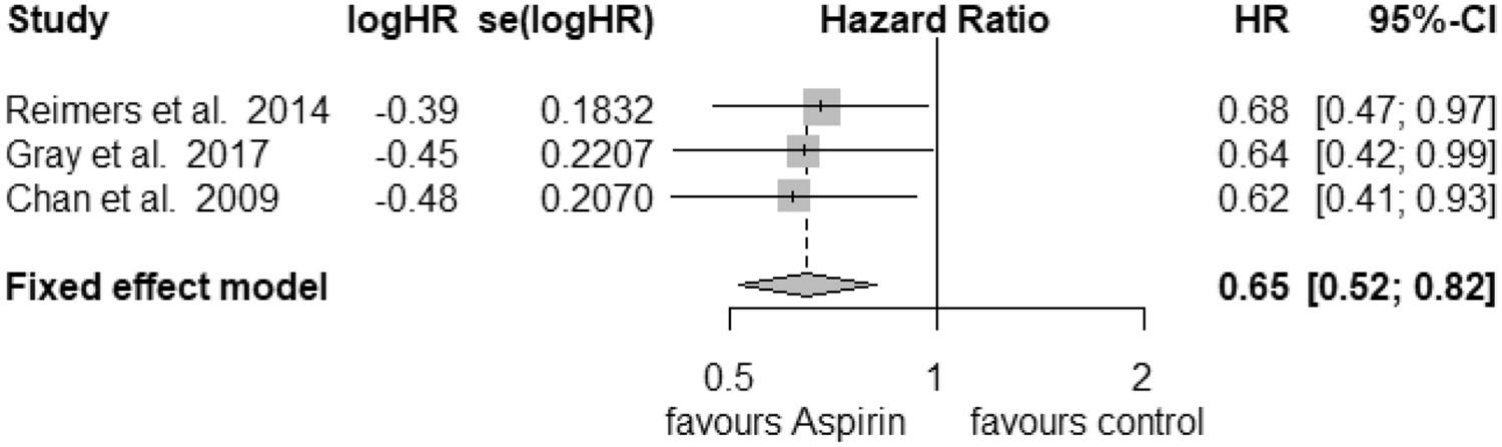
Postdiagnosis aspirin use, high PTGS2

**Fig. 7:**
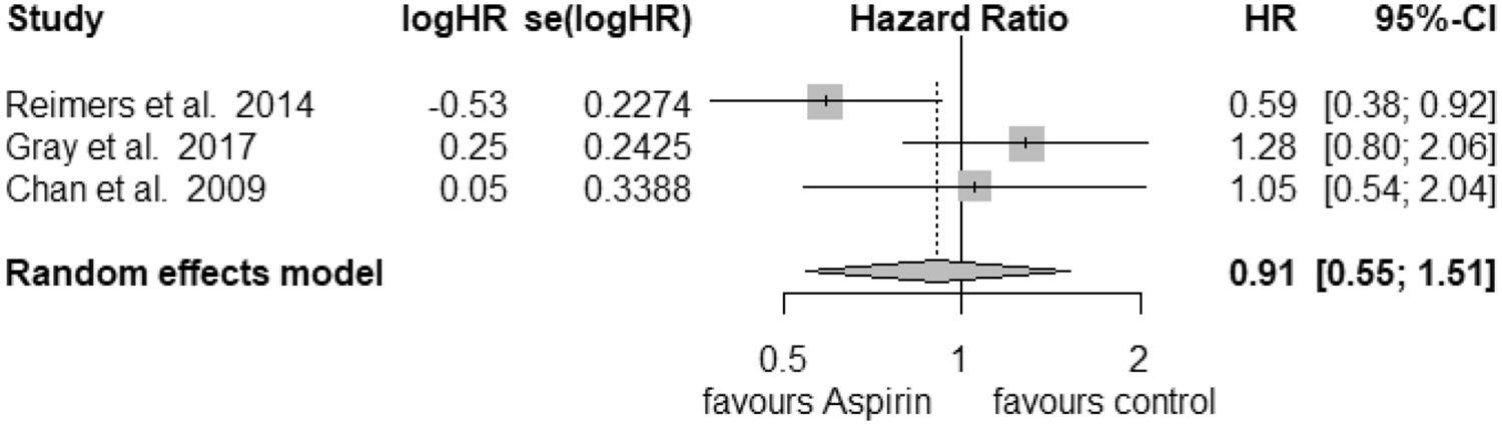
Postdiagnosis aspirin use, low PTGS2

Only 3 studies were recruited for further analysis of PTGS2 (COX-2) status in the postdiagnosis aspirin use group. For high expression of PTGS2 (Fig. 6) we calculated a reduction in mortality with a pooled HR of 0.65 (95% CI: 0.52–0.82) with a heterogeneity of *I*^2^ = 0% (tau^2^ = 0, *Q* = 0.12, *p* = 0.94). Here, we also used the fixed-effects model, since no heterogeneity was found. No publication bias was assessed (*t* = − 1.31, *p* = 0.41). Low expression of PTGS2 (Fig. 7) was associated with an HR of 0.91 (95% CI: 0.55–1.51) and a heterogeneity of *I*^2^ = 65% (tau^2^ = 0.1309, *Q* = 5.75, *p* = 0.06). Likewise, no publication bias was found (*t* = 0.42, *p* = 0.74).

### Side effects of aspirin use

We also examined the relevant side effects, especially bleeding, which were described in the selected studies. In total, only a few publications commented on this issue. Frouws et al. (Frouws et al. 2017) demonstrated that low-dose aspirin therapy for cardiovascular prevention doubled the incidence of gastric bleeding. In their study, they reported that 1 or 2 per thousand individuals are likely to have a gastric bleeding event per year and even up to 7 per thousand for patients older than 80 years. Giampieri et al. (Giampieri et al. 2017) did not observe intestinal bleeding in either the group of patients receiving aspirin or in the control group.

## Discussion

The results of our meta-analysis indicate that aspirin use after a diagnosis of CRC was associated with better overall survival and in contrast to the meta-analysis of Li et al. (Li et al. 2015) even better CRC-specific survival than standard therapy. For recurrence-free survival, aspirin use after diagnosis seems to have a positive effect on patient’s outcome. However, we must remark that only three studies presented usable data. The analysis of overall survival regarding PIK3CA gene expression favours mutated tumours. In contrast, patients with wild-type PIK3CA status did not profit from postdiagnosis aspirin use. Additionally, PTGS2 (COX-2) status seems to be another important factor of the patient’s outcome. High expression of PTGS2 is associated with better overall survival than lower expression. For prediagnosis aspirin use, we calculated the same HR for both CRC-specific survival and overall survival. We did not find a survival benefit of patients with CRC.

Until now, most studies have considered the effect of aspirin on the outcome of patients with CRC as one large group. One strength of our meta-analysis is the analysis of different genotypes. They are of special interest, since it is important to know for which group of patients an aspirin-guided therapy of CRC should be considered. Few articles studied different genes in more detail. Looking at our study, it appears that for some groups of patients, the benefit of therapy with aspirin outweighs the risk, whereas others would only be at risk of gastrointestinal bleeding. Thus, it appears that a closer look at gene expression requires further research to personalise therapy for each patient.

Furthermore, we used the Newcastle Ottawa Scale (Stang 2010) to assess study quality. It classifies the selected studies based on objective criteria, though a subjective influence remains. Most of the studies—apart from the two case-control studies—were of high quality, which is another advantage of this meta-analysis.

Due to the small number of studies, for each subgroup analysis, we presented estimated *I*^2^, tau^2^ and *Q* values to increase the transparency of our calculations. No publication bias was found in this meta-analysis. Our study was influenced by high heterogeneity of the selected studies. In contrast, PIK3CA mutation status, high expression of PTGS2 and recurrence-free survival were not biased by heterogeneity. For those three studies, the estimated *I*^2^ was 0%, so we presented the corresponding forest plots based on the fixed-effects model. This supports the thesis that patients in those groups might benefit from therapy with aspirin in addition to standard care for CRC.

We must admit that we did not find all data of interest in each study. For example, aspirin dosage is of prime importance for potential adjuvant therapy, but not all the included studies described appropriate details on medication. In most cases, there was no information given about how many patients took aspirin. The aspirin dosages varied widely, between 75 and 325 mg. However, it may be of importance for CRC patients’ survival whether they have low-dose (< 300 mg) or high-dose aspirin therapy. Furthermore, higher dosages could be associated with more gastrointestinal bleeding events. In our research, we found very little information about side effect. More randomised studies are needed to find the optimal dosage for an adjuvant use and to learn more about potential side effects.

Another important factor in CRC patient outcomes is the cancer stage. Most of the selected studies used patients with stages I–IV. Only a few limited their inclusion criteria for certain groups of stage. For our analysis, we used multivariable results which were adjusted for stage in 20 of the 27 studies. Nevertheless, survival depends on the stage of CRC and it is obvious that a lower stage may be associated with better survival. In conclusion, further research on aspirin use in patients with different stages of severity is needed.

In our meta-analysis, we investigated PIK3CA and PTGS2 (COX-2). For both subgroup analyses, we must point out that the number of eligible studies was relatively small. Unfortunately, we did not find enough studies to analyse other genes such as Kirsten rat sarcoma viral oncogene (KRAS) or v-raf murine sarcoma viral oncogene homolog B (BRAF) which are of interest to research on CRC. Frouws et al. (Frouws et al. 2017) described a benefit for overall patient survival with wild-type-BRAF tumours (RR = 0.60 (95% CI: 0.44–0.83) and no association between KRAS mutation status and aspirin use after diagnosis of CRC.

Our review almost exclusively involved cohort (and two case-control studies), which provide a lower level of evidence. Cohort studies are more credible than case-control studies. Nevertheless, a few randomised controlled trials have been published regarding the concern of postdiagnosis aspirin use as adjuvant therapy for CRC. Fortunately, several studies are in process and will present further knowledge on the well-known drug acetylsalicylic acid.

We would like to emphasise that so far aspirin has only been considered as primary prevention in most studies. This has often been critically debated. In our work, we show that even after a confirmed diagnosis of CRC, there is still a therapeutic benefit with aspirin. Accordingly, this approach of tertiary prevention should not be underestimated.

## Conclusion

In summary we consistently found that aspirin appears to have a favourable effect on the outcome of patients with colorectal cancer. Thus, it could be considered as a potential therapeutic approach in these patients.

## Supplementary Information

Below is the link to the electronic supplementary material.

Supplementary file1 (PDF 342 KB)

Supplementary file2 (PDF 9 KB)

Supplementary file3 (PDF 9 KB)

Supplementary file4 (PDF 8 KB)

## Data Availability

The datasets generated during or analysed during the current study are available from the corresponding author on reasonable request.

## Abbreviations

n.a: Not available
CRC: Colorectal cancer
CC: Colon cancer
RC: Rectal cancer
Pre: Prediagnosis aspirin use
Post: Postdiagnosis aspirin use
PIK3CA: Phosphatidylinositol-4, 5-bisphosphate 3-kinase catalytic subunit alpha
KRAS: Kirsten rat sarcoma viral oncogene
BRAF: V-raf murine sarcoma viral oncogene homolog B
PTGS2: Prostaglandin-endoperoxide synthase 2
HLA class 1: Human leukocyte antigen class 1
CIMP: CpG island methylator phenotype
LINE-1: Long interspersed nuclear element
Phosphorylated AKT: Phosphorylated protein kinase B
CD274: Cluster of differentiation 274 (= programmed cell death 1 ligand 1
