# Supplementary material for: Meta-analysis of aspirin-guided therapy of colorectal cancer
Source: J Cancer Res Clin Oncol. 2022 Feb 16;148(6):1407–17. doi: 10.1007/s00432-022-03942-1 (PMC9114035; doi:10.1007/s00432-022-03942-1)
Supplement: Supplementary file 1 — Supplementary file1 (PDF 342 KB) [file 432_2022_3942_MOESM1_ESM.pdf]

Study	Year	Study design	Data source	sample size	Sex	Number of Adjust-ments	age-adj.	sex-adj.	grade-adj.	stage-adj.	Gene	Dose (mg)	pre-/post-diagnosis Aspirin use	Cancer type	Country	Age	Stage	Outcome	Study quality
Bains et al. [23]	2016	observational, population-based, retrospective cohort study	CRN + Norwegian Prescription Database (NorPD)	23162	3	11	x	x	-	x	-	75/160	post	CRC	Norway	71.5	I-IV	all-cause deaths: 9289, CRC-specific deaths: 6533	9
Bastiaannet et al. [24]	2012	observational population-based study	ECR + PHARMO record linkage systems 1998–2007	4481	3	9	x	x	x	x	-	80/30	post	CRC	Nether-lands	69	I-IV	n.a.	8
Cardwell et al. [17]	2014	Case–Control Analysis of a population-based cohort	NCDR + CPRD + Office of National Statistics death registration data (ONS)	12868	3	6	-	-	-	-	-	25 (0.3%)/ 75 (98.5%)/ >300 (1.2%)	pre + post	CRC	UK	n.a.	I-IV	all-cause deaths: 2214, CRC-specific deaths: 1559	4
Chan et al. [25]	2009	prospective cohort study	NHS + HPFS	1279	3	12	x	x	x	x	-	325	pre + post	CRC	US	65.0	I-III	all-cause deaths: 480, CRC-specific deaths: 222	7

Meta-analysis of aspirin-guided therapy of colorectal cancer, Journal of Cancer Research and Clinical Oncology,
J. C. Mädge (corresponding author, Department of Medical Statistics, Computer Sciences and Data Sciences, Jena University Hospital, 07743 Jena, Germany, johannamaedge@t-online.de), A. Stallmach, L. Kleebusch, P. Schlattmann

Domingo et al. [5]	2013	subanalysis of RCT	VICTOR trial	896	3	6	x	x	x	-	PIK3CA	<100	post	CRC	UK	64.6	II-III	all-cause deaths: 395	8
Frouws et al. [20]	2017	retrospective cohort study	ECR + PHARMO Database network 2002-2008	599	3	5	x	-	x	x	BRAF, KRAS	80-100	post	CC	Nether-lands	<65: 31.6%, 66-74: 31.6%, >75: 36.9%	I-IV	all-cause deaths: 267	8
Goh et al. [26]	2014	retrospective cohort study	SGH + NCC	726	3	7	x	-	x	x	-	100	pre + post	CRC	Singa-pore	65	I-III	CRC-specific deaths: 181	8
Gray et al. [27]	2017	Population-based cohort study	Northern Ireland Cancer Registry	680	3	7	x	x	x	x	PTGS2, PIK3CA	75	post	CRC	Norther n Ireland	70.3	II-III	all-cause deaths: 299, CRC-specific deaths: 212	8
Hamada et al. [28]	2017	retrospective analysis of 2 prospective cohort studies	NHS + HPFS (1980/86-2008)	617	3	7	x	-	-	x	CD274	81/325	post	CRC	US	68.6	I-IV	all-cause deaths: 325, CRC-specific deaths: 118	6
Hua et al. [29]	2017	Population-based prospective study	CCFR study centers 1997 - 2008	2419	3	5	-	x	-	x	KRAS, BRAF	n.a.	pre + post	CRC	US	54	I-IV	all-cause deaths: 381, CRC-specific deaths: 100	8
Liao et al. [30]	2012	prospective cohort study	Nurses' Health Study + Health Professionals Follow-up Study	964	3	15	x	x	-	x	PIK3CA, KRAS, BRAF, PTGS2, CIMP, LINE-1,	325	post	CRC	US	68.0	I-IV	all-cause deaths: 395, CRC-specific deaths: 190	6

											phosphory-lated AKT								
McCowan et al. [31]	2013	retrospective observational population cohort study	Health Informatics Centre 1997-2006 Scotland	2990	3	5	x	x	-	x	-	75/300	pre + post	CRC	UK	73	I-IV	all-cause deaths: 1998, CRC-specific deaths: 1021	9
Ng et al. [32]	2015	prospective, observational study	CALGB 89803	799	3	11	x	x	x	-	-	n.a.	post	CC	US	n.a.	III	all-cause deaths: 156	6
Reimers et al. [33]	2014	retrospective cohort study	ECR + PHARMO Database network 2002-2008	999	3	7	x	x	x	x	PTGS2, PIK3CA, HLA Class I	75-325	post	CC	Netherlands	<65: 34.2%, 66-74: 30.4%, >75: 35.4%	I-IV	all-cause deaths: 465	8
Walker et al. [34]	2012	retrospective cohort study	General Practice Research Database	13944	3	5	x	x	-	-	-	75/>75	pre + post	CRC	UK	Aspirin non-user: 74.5; user: 68.3	I-IV	all-cause deaths: 5358	8
Coghill et al. (Gut) [35]	2011	retrospective cohort study	Seattle Colon CFR + population based Puget Sound SEER registry 1997-2002	1737	3	7	x	x	-	-	-	n.a.	pre	CRC	US	20-74; <50: 16.4%, 50-59.9: 27.2%, 60-69.9: 35.6%, >70: 20.7%	I-IV	all-cause deaths: 707, CRC-specific deaths: 262	8

Coghill et al. (British Journal of Cancer) [36]	2011	cohort study	Surveillance, Epidemiology and End Results cancer registry SEER + National Death Index 1997-2002	1051	1	6	x	-	-	x	-	n.a.	pre	CRC	US	Never/ever NSAID use: <50: 11.6%/11.4 %, 50-59: 28.6%/23.0 %, 60-69: 34.7%/39.3 %, >70: 25.0%/26.2 %	I-IV	all-cause deaths: 371, CRC-specific deaths: 274	9
Coghill et al. [37]	2012	cohort study	Women's Health Initiative	160143	1	9	x	-	-	-	-	<200- >325mg	pre	CRC	US	50-54: 13.36%, 55-59: 19.82%, 60-69: 44.9%, 70-79: 21.92%	I-IV	all-cause deaths: 15608, CRC-specific deaths: 492	7
Din et al. [18]	2010	case-control study	SOCCS	2259	3	4	X	X	-	x	-	75	pre	CRC	UK	62.2 (cases)	I-IV	all-cause deaths: 670, CRC-specific deaths: 561	5
Giampieri et al. [21]	2017	retrospective observational analysis	D. of Medical Oncology of the Polytechnic University of the Marche, Italy +	66	3	7	x	X	-	-	KRAS, BRAF	100	pre	CRC	Italy	62	I-III	all-cause deaths: 66	8

			D. of Medical Oncology of the University of Cagliari, Italy																
Hippisley-Cox et al. [19]	2017	cohort study	UK QResearch database -> derivation cohort (+PHE and national cancer registry)	44145	3	19	x	-	x	x	-	n.a.	pre	CRC	UK	Women deriva-tion cohort: 72.5; Men deriva-tion cohort: 70.9	I-IV	all-cause deaths: 26887, CRC-specific deaths: 13588 (derivation cohort)	8
Kim et al. [38]	2015	retrospective study	Severance Hospital	686	3	8	x	x	-	x	-	n.a.	pre	CRC	Korea	Aspirin non-user: 57.55; predia-gnostic aspirin user: 66.3	III	n.a.	8
Kothari et al. [39]	2015	retrospective analysis of prospective data	MCC in the US + RMH in Australia	1487	3	7	x	-	-	x	PIK3CA	81-325	pre	CRC	US, Austra-lia	Aspirin non-user: 70; aspirin user: 74	I-IV	n.a.	8
Murphy et al. [40]	2017	retrospective analysis of prospective data	ACCORD	488	3	5	x	-	-	x	PIK3CA	>75	post	CC	Austra-lia	72	II	all-cause deaths (PIK3CA-M.): 17; all-cause deaths (PIK3CA-WT.): 80	8

Zell et al. [41]	2009	prospective cohort study	California Teachers Study	621	1	6	x	-	-	x	-	n.a.	pre	CRC	US	Regular NSAID Use: 65.0, none: 63.2	I-IV	all-cause deaths: 222, CRC-specific deaths: 145	7
Zanders et al. [42]	2015	cohort study	ECR + PHARMO Database network 1998-2011	1043	3	15	x	x	-	x	-	<100	post	CRC	Nether-lands	73.2	I-IV	all-cause deaths: 494	9
Restivo et al. [43]	2015	cohort study	Colorectal Surgery Center of the University of Cagliari (Italy)	241	3	7	x	x	x	x	-	100	post	RC	Italy	65	II-III	n.a.	8

Explanation: Sex: 1 = women, 2 = men, 3 = both,

Abbreviations: n.a.= not available; CRC = colorectal cancer, CC = colon cancer, RC = rectal cancer, pre = prediagnosis Aspirin use, post = postdiagnosis Aspirin use, PIK3CA = Phosphatidylinositol-4,5-Bisphosphate 3-Kinase Catalytic Subunit Alpha, KRAS = Kirsten rat sarcoma viral oncogene, BRAF = v-raf murine sarcoma viral oncogene homolog B, PTGS2 = prostaglandin-endoperoxide synthase 2, HLA class 1 = human leukocyte antigen class 1, CIMP = CpG island methylator phenotype, LINE-1 = long interspersed nuclear element, phosphorylated AKT = phosphorylated protein kinase B, CD274 = Cluster of differentiation 274 (= Programmed cell death 1 ligand 1), adj. = adjusted, CRN = Cancer Registry of Norway, ECR = Eindhoven Cancer Registry, NCDR = National Cancer Data Repository, CPRD = Clinical Practice Research Datalink, ACCORD = Australian Comprehensive Cancer Outcomes and Research Database, NHS = Nurses' Health Study, MCC = Moffitt Cancer Center, RMH = Royal Melbourne Hospital, HPFS = Health Professionals Follow-up Study, SGH = Singapore General Hospital, NCC = National Cancer Centre Singapore, SOCCS = Study of Colorectal Cancer in Scotland, D. = Department
