# Supplementary material for: Meta-analysis of aspirin-guided therapy of colorectal cancer
Source: J Cancer Res Clin Oncol. 2022 Feb 16;148(6):1407–17. doi: 10.1007/s00432-022-03942-1 (PMC9114035; doi:10.1007/s00432-022-03942-1)

Meta-analysis of aspirin-guided therapy of colorectal cancer, Journal of Cancer Research and Clinical Oncology,
J. C. Mädge (corresponding author, Department of Medical Statistics, Computer Sciences and Data Sciences,
Jena University Hospital, 07743 Jena, Germany, johannamaedge@t-online.de), A. Stallmach, L. Kleebusch, P. Schlattmann

Study	logHR	se(logHR)	HR	95%−CI
Kothari et al. 2015	−0.42	0.3889	0.66	[0.31; 1.41]
Din et al. 2010	0.03	0.1284	1.03	[0.80; 1.32]
Hippisley−Cox et al. (Women) 2017	−0.15	0.0574	0.86	[0.77; 0.96]
Hippisley−Cox et al. (Men) 2017	−0.26	0.0339	0.77	[0.72; 0.82]
Coghill et al. (British Journal of Cancer) 2011	0.13	0.2047	1.14	[0.76; 1.70]
Coghill et al. (Gut) 2011	−0.27	0.1126	0.76	[0.61; 0.95]
Zell et al. 2009	−0.48	0.2161	0.62	[0.41; 0.95]
Cardwell et al. 2014	0.03	0.0654	1.03	[0.91; 1.17]
McCowan et al. 2013	−0.04	0.0726	0.96	[0.83; 1.11]
Chan et al. 2009	0.05	0.1401	1.05	[0.80; 1.38]
Goh et al. 2014	0.57	0.2485	1.76	[1.08; 2.86]
Hua et al. 2017	−0.80	0.4525	0.45	[0.19; 1.09]
Coghill et al. 2012	−0.08	0.1242	0.92	[0.72; 1.17]
**Random effects model**			**0.91**	**[0.82; 1.01]**

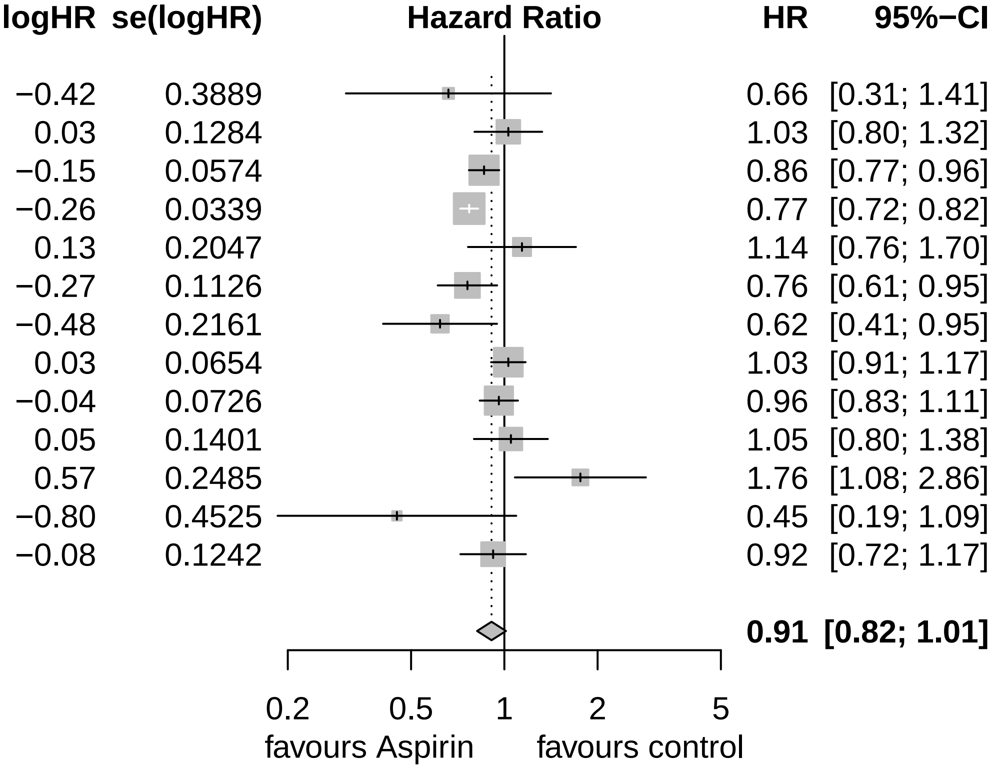

Supplement: Supplementary file 2 — Supplementary file2 (PDF 9 KB) [file 432_2022_3942_MOESM2_ESM.pdf]
