# Supplementary material for: Meta-analysis of aspirin-guided therapy of colorectal cancer
Source: J Cancer Res Clin Oncol. 2022 Feb 16;148(6):1407–17. doi: 10.1007/s00432-022-03942-1 (PMC9114035; doi:10.1007/s00432-022-03942-1)

Meta-analysis of aspirin-guided therapy of colorectal cancer, Journal of Cancer Research and Clinical Oncology,
J. C. Mädge (corresponding author, Department of Medical Statistics, Computer Scienes and Data Sciences,
Jena University Hospital, 07743 Jena, Germany, johannamaedge@t-online.de), A. Stallmach, L. Kleebusch, P. Schlattmann

Study	logHR	se(logHR)	HR	95%−CI
Kothari et al. 2015	−0.05	0.2829	0.95	[0.55; 1.65]
Din et al. 2010	0.11	0.1132	1.12	[0.90; 1.40]
Giampieri et al. 2017	−0.84	0.2653	0.43	[0.26; 0.72]
Hippisley−Cox et al. Women 2017	−0.11	0.0549	0.90	[0.81; 1.00]
Hippisley−Cox et al. Men 2017	−0.20	0.0314	0.82	[0.77; 0.87]
Zell et al. 2009	−0.30	0.1631	0.74	[0.54; 1.02]
McCowan et al. 2013	−0.01	0.0499	0.99	[0.90; 1.09]
Walker et al. 2012	0.04	0.0374	1.04	[0.97; 1.12]
Chan et al. 2009	−0.07	0.0952	0.93	[0.77; 1.12]
Hua et al. 2017	−0.12	0.1805	0.89	[0.62; 1.27]
**Random effects model**			**0.91**	**[0.82; 1.01]**

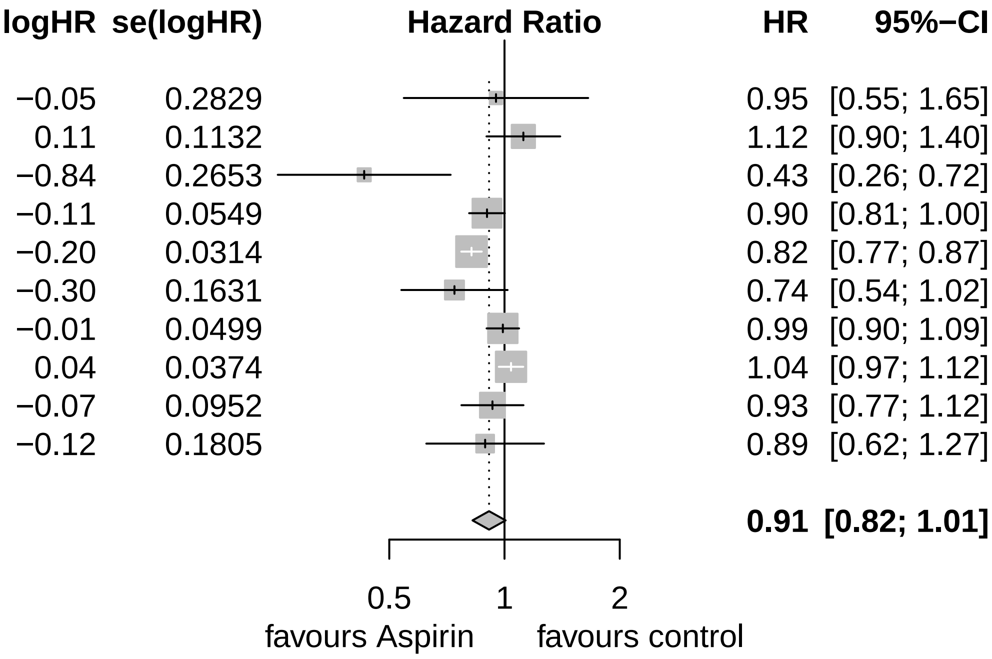

Supplement: Supplementary file 3 — Supplementary file3 (PDF 9 KB) [file 432_2022_3942_MOESM3_ESM.pdf]
