# Supplementary material for: Meta-analysis of aspirin-guided therapy of colorectal cancer
Source: J Cancer Res Clin Oncol. 2022 Feb 16;148(6):1407–17. doi: 10.1007/s00432-022-03942-1 (PMC9114035; doi:10.1007/s00432-022-03942-1)

Meta-analysis of aspirin-guided therapy of colorectal cancer, Journal of Cancer Resarch and Clinical Oncology,
J. C. Mädge (corresponding author, Department of Medical Statistics, Compuer Sciences and Data Sciences,
Jena University Hospital, 07743 Jena, Germany, johannamaedge@t-online.de), A. Stallmach, L. Kleebusch, P. Schlattmann

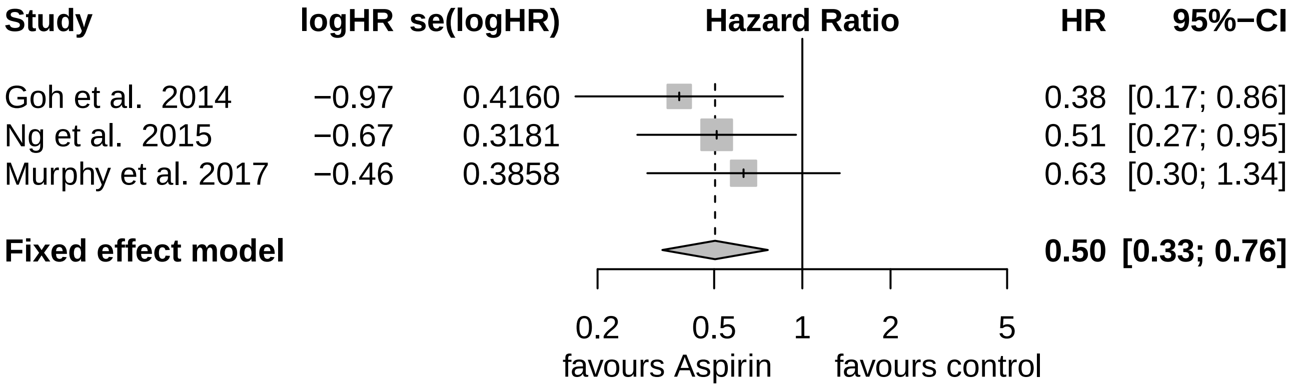

Supplement: Supplementary file 4 — Supplementary file4 (PDF 8 KB) [file 432_2022_3942_MOESM4_ESM.pdf]
